# Distinct Electrification Regimes at Solid–Liquid Interfaces Revealed by Water Isotope Substitution

**DOI:** 10.1021/acs.jpcc.6c04134

**Published:** 2026-07-28

**Authors:** Mesude Z. Arkan, Luis Bartolomé, Raivis Egli̅tis, Manuel Brinker, Eder Amayuelas, Patrick Huber, Mirosław Chorążewski, Simone Meloni, Andris Šutka, Yaroslav Grosu

**Affiliations:** † Institute of Chemistry, 49568 University of Silesia, Katowice 40-006, Poland; ‡ Centre for Cooperative Research on Alternative Energies (CIC energi GUNE), Basque Research and Technology Alliance (BRTA), Alava Technology Park, Albert Einstein 48, Vitoria-Gasteiz 01510, Spain; § Institute of Physics and Materials Science, Faculty of Natural Sciences and Technology, 468614 Riga Technical University, P. Valdena Street 3/7, Riga LV-1048, Latvia; ∥ 38987 Hamburg University of Technology, Institute for Materials and X-Ray Physics, Denickestr. 15, Hamburg 21073, Germany; ⊥ Centre for X-ray and Nano Science CXNS, Deutsches Elektronen-Synchrotron DESY, Notkestr. 85, Hamburg 22607, Germany; # Centre for Molecular Water Science CMWS, Deutsches Elektronen-Synchrotron ​DESY, Notkestr. 85, Hamburg 22607, Germany; ¶ Dipartimento di Scienze Chimiche e Farmaceutiche Università degli Studi di Ferrara, Via Luigi Borsari 46, Ferrara I-44121, Italy

## Abstract

Solid–liquid contact electrification is widely studied using diverse experimental geometries that are often implicitly assumed to probe equivalent interfacial processes. Here, we directly test this assumption by comparing immersion–emersion, droplet-based contact, and pressure-driven intrusion–extrusion electrification using the same hydrophobic nanoporous silicon monolith and isotopically substituted liquids (H_2_O and D_2_O). Despite identical surface chemistry, isotopic substitution produces qualitatively different electrical responses depending on the wetting regime. Immersion–emersion experiments show polarity inversion between H_2_O and D_2_O, while droplet measurements reveal distinct charge evolution and periodic opposite-polarity events. In contrast, forced nanopore intrusion preserves polarity but strongly enhances electrical output for D_2_O. These results demonstrate that wetting of surface nanoroughness during droplet, vs immersion–emersion, vs complete nanopores wetting upon intrusion–extrusion correspond to fundamentally different electrification states. By using isotope substitution as a controlled probe, this work establishes a general framework for disentangling protocol-dependent effects in solid–liquid contact electrification and triboelectric energy conversion.

## Graphical abstract

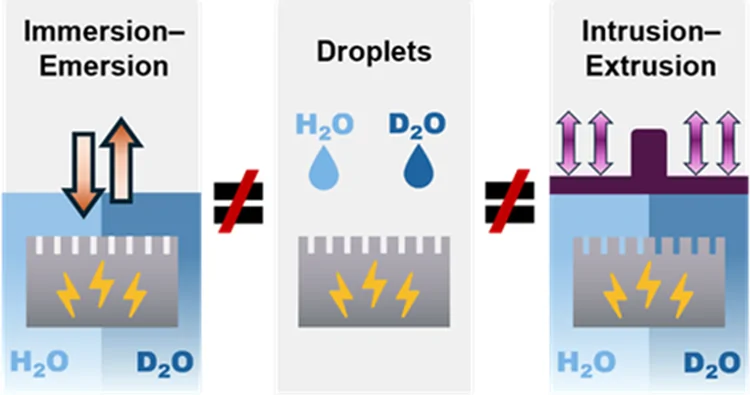

## 1 Introduction

Contact electrification (CE) is a pervasive interfacial phenomenon underpinning a wide spectrum of natural processes and technological applications, ranging from atmospheric electricity, aerosol dynamics, and particle adhesion to sensing, actuation, and mechanical-to-electrical energy conversion.^1–3^ In modern literature, contact electrification (triboelectrification) is understood as a collective interfacial phenomenon that may arise from several coupled processes occurring when two materials come into contact, including electron transfer between surface states,^4^ ion transfer mediated by adsorbed species or mobile surface ions,^5^ material transfer,^6^ and interfacial chemical reactions at the interface.^7–9^ In recent years, the rapid development of triboelectric nanogenerators (TENGs) has transformed CE from a scientific curiosity into a central functional principle for self-powered electronics, distributed sensors, and energy harvesting systems, stimulating an unprecedented volume of experimental and applied research.^10–12^ At the same time, it has become increasingly clear that, particularly for solid–liquid interfaces, CE cannot be treated as a single, universal process. While numerous studies have reported reproducible electrical outputs across a wide variety of material–liquid combinations, the interpretation of these signals remains highly system- and protocol-dependent.^13–15^ Rather than converging toward a unified microscopic picture, the literature reveals that CE at liquid interfaces is strongly influenced by interfacial geometry, wetting dynamics, and confinement, often without these factors being explicitly disentangled.^13,16,17^

This challenge is particularly evident in solid–liquid TENGs (SL-TENGs), where electrification has been investigated using conceptually distinct experimental configurations, including liquid droplets impacting or sliding on solids, periodic immersion–emersion of solids in bulk liquids, and pressure-driven intrusion–extrusion of liquids into nanoporous materials.^18–20^ These configurations are sometimes discussed within a common conceptual framework, electronic and/or ionic charge transfer between the liquid and the solid, implicitly assuming that they probe equivalent interfacial charge-generation processes. However, such an assumption remains largely untested. Each configuration imposes fundamentally different constraints on wetting kinetics, interfacial area evolution, molecular organization of the liquid at the solid surface and on the mechanical energy delivered to the interface.

More recently, isotope-sensitive electrical responses have been reported in water-contact sensing and energy-conversion devices, highlighting that replacing H_2_O with D_2_O can measurably alter interfacial electrification signals even when chemical composition and macroscopic properties remain nearly identical.^21^ Nevertheless, comparative studies that systematically examine how the same solid responds to chemically similar but isotopically distinct liquids, such as H_2_O and D_2_O, across these different electrification regimes are notably scarce.^14,22,23^ Existing studies have primarily explored isotope effects through electrokinetic proxies such as zeta potential, electrophoretic mobility, or ice–water interfacial charging, where measurable differences are attributed to altered hydrogen-bond networks, proton/deuteron dynamics, and interfacial structure.^24–26^ However, these studies typically focus on a single experimental configuration and do not address whether the observed isotope sensitivity is intrinsic to charge-generation processes or instead reflects differences in wetting dynamics and interfacial geometry.

In this work, rather than introducing a new hypothesis for charge transfer and contact electrification, we provide a unified experimental comparison of three distinct solid–liquid electrification modes: (i) immersion–emersion, (ii) intrusion–extrusion, and (iii) droplet-based contact, using the same hydrophobic nanoporous solid and directly contrasting H_2_O vs D_2_O. The resulting observations demonstrate that wetting of hydrophobic surface nanoroughness during droplet contact or immersion–emersion differs qualitatively from complete forced wetting of the same roughness under pressure, with opposite charging depending on the experiment, despite the same material being used throughout. This underscores that distinct charging mechanisms may operate across the three configurations in the CE experiments.

From a methodological perspective, the H_2_O/D_2_O comparison offers a unique and underutilized experimental advantage for solid–liquid electrification studies. Because H_2_O and D_2_O share the same electronic structure and differ primarily in nuclear mass, hydrogen-bond strength, and associated dynamics, isotope substitution enables selective perturbation of interfacial organization and relaxation processes without altering surface chemistry or device architecture. When applied across distinct electrification modes, this approach allows one to test whether different experimental protocols (in particular, wetting only surface nanoroughness vs complete intrusion of the pores) truly probe equivalent interfacial states.

In this work, the combination of three electrification regimes with systematic H_2_O vs D_2_O comparison reveals that wetting of surface nanoroughness under ambient immersion, transient wetting during droplet contact, and pressure-driven wetting of the same roughness under nanoconfinement are fundamentally nonequivalent from an electrification standpoint. By enabling a unified experimental comparison within a single material platform, this approach provides the CE community a robust framework for distinguishing protocol-dependent effects from material-intrinsic behavior and for interpreting data in solid–liquid contact electrification.

## 2 Materials and Methods

### 2.1 Materials

The hydrophobic nanoporous silicon monoliths used in this work were produced using the protocol described in our previous article. Porous silicon was created using an electrochemical etching process on p-type, single-crystal silicon wafers with a resistivity of 0.01–0.02 Ω·cm, (100) crystal orientation, a thickness of 525 ± 25 μm, and polished on one side. The wafer was placed in an electrochemical cell filled with a 2:3 volume ratio of hydrofluoric acid and absolute ethanol. A current was passed between a platinum counter electrode and the silicon wafer. To create a porous silicon layer, a current density of 80 mA·cm-2 was used. The etching process lasted for 10 min. Following the initial step, a thin layer of silicon dioxide (SiO_2_) approximately 1 nm thick was formed on the inner surfaces of the pores by immersing the porous silicon samples in hydrogen peroxide (H_2_O_2_) for 20 h. To make the surface hydrophobic, the monolith was vacuum-dried overnight at 120 °C, then placed in a 1.1 M ethanolic ammonia solution at 50 °C and stirred for 30 min. Fluorinated C8 alkyl silane (1H,1H,2H,2H-perfluorooctyltrichlorosilane, or PFOTCS) was grafted onto the surface by adding it dropwise in excess—at a concentration of 5 molecules per nm^2^ of the porous surface—followed by continuous stirring for 120 h. After 5 days, the monolith was rinsed three times with ethanol and dried overnight at 70 °C. Finally, two surface-modified silica sheets were joined using silver epoxy, with a conductive wire positioned between them to ensure that their hydrophobic surfaces remained exposed (see Figure 1). Apparent contact-angle measurements were performed for H_2_O and D_2_O on the hydrophobic silicon monolith surface, showing comparable macroscopic wettability for both liquids (see Supp. Inf. Section S1).

**Figure 1:**
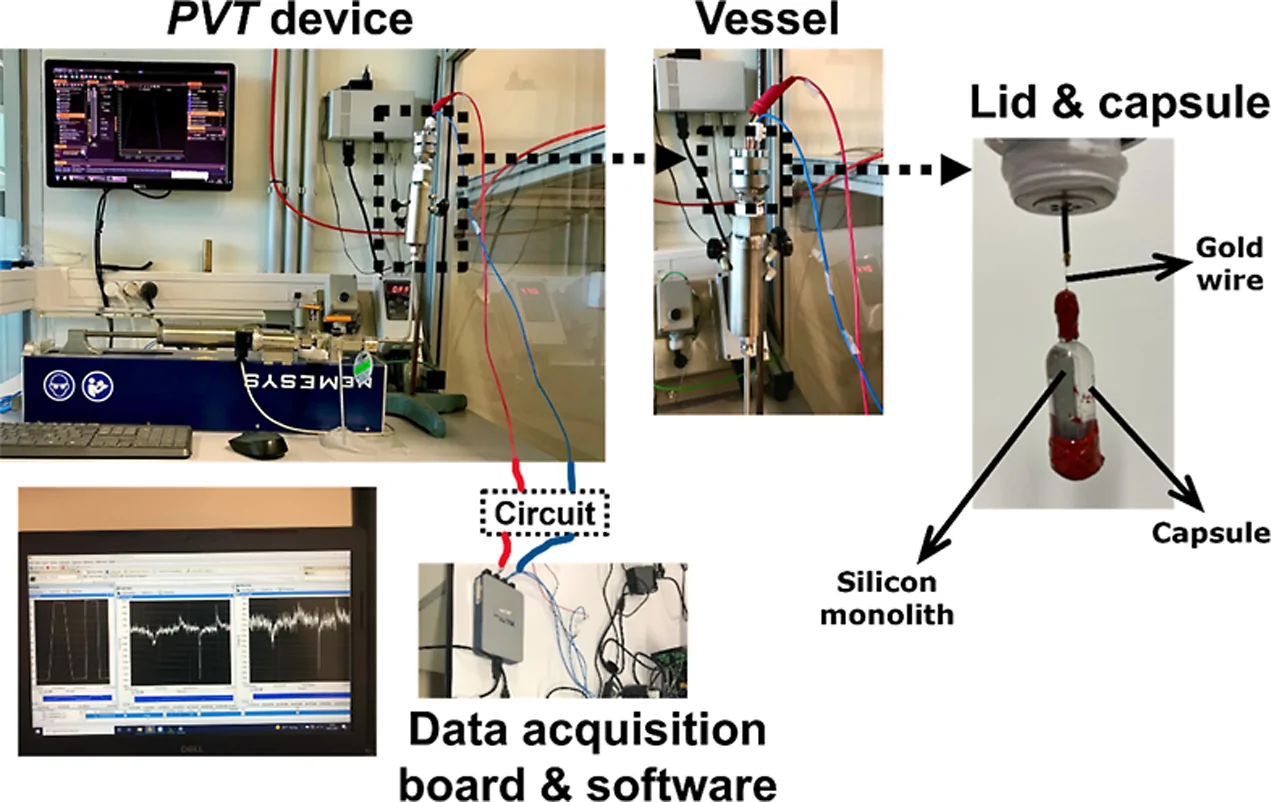
PVT device, components and peripheral units. PVT device with all the components, zoom of the metallic vessel where the samples are located, with the lid to close it and pass-through wires to connect to the data acquisition (DAQ) system with the corresponding circuit (see Figure 5a). An encapsulated silicon monolith sample connected to the lid using a gold wire attached using conductive silver paste.

The nanoporous silicon monolith used in this work exhibited a characteristic pore diameter of 15.4 nm (see Supp. Inf. Section S2), a porous layer thickness of 31.8 μm, a surface area of 1.982 m^2^, and a total pore volume of 0.0086 cm^3^, as determined from N_2_ physisorption measurements using the BJH model; further details regarding surface characterization are provided in our previous paper.^27^ The pH of the H_2_O and D_2_O used in this work was ∼7.0. Distilled water Type II (analytical grade) and commercially supplied deuterium oxide (D_2_O; Sigma-Aldrich, CAS: 7789–20–0; 99.8 atom % D) were used in all experimental methods. The measured electrical conductivity was 2.0–2.5 μS/cm after exposure of the liquids to ambient air (this reflects the practical experimental conditions).

### 2.2 Immersion-Emersion Triboelectric Experiments

For the immersion-emersion tests, an aluminum foil (2 × 7.5 cm) was cut and used as the counter electrode, with a conductive cable attached to the back. The other end of the cable was connected to an oscilloscope for voltage measurement. The nanoporous silicon monolith was used as the working electrode, connected with a conductive cable to the second input channel of the oscilloscope. The measurements were performed in a glass tank with dimensions 6.5 × 1.3 × 1.6 cm (H × W × L), filled with 10 mL of liquid, either water (H_2_O) or heavy water (D_2_O). The counter electrode was placed vertically inside the tank, fully immersed in the liquid. The working electrode, i.e., the nanoporous silicon monolith, was then periodically immersed and emersed in the liquid, as shown in Figure 3a. During this motion, the instantaneous voltage output of the system was recorded using a Rigol DHO4204 oscilloscope with an internal resistance of 10 MΩ. The proper operation of the experimental setup was validated using a PTFE-coated aluminum electrode with H_2_O; the corresponding results are provided in the Supp. Inf. Section S3.

**Figure 2:**
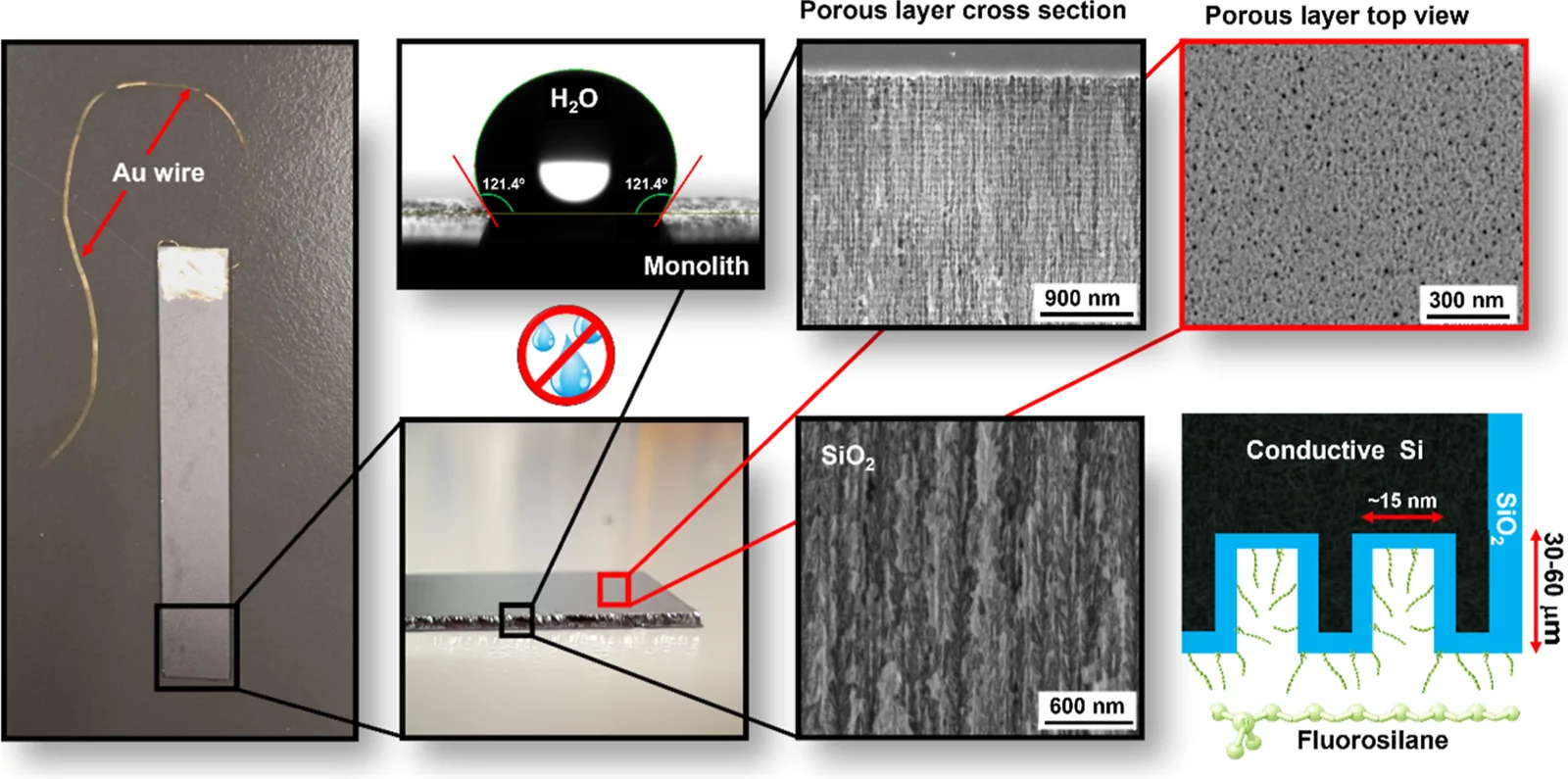
Structure and surface properties of the hydrophobic nanoporous silicon monolith used for solid–liquid contact electrification experiments. Left: photograph of the macroscopic monolith electrode. Center: optical image of the monolith surface and representative sessile water droplet demonstrating strong hydrophobicity (contact angle ≈121°), indicating that liquid does not spontaneously penetrate the nanopores. Right: electron microscopy characterization of the porous silicon layer. Cross-sectional view shows vertically aligned nanopores extending through the porous layer, while the top view reveals the nanoporous morphology. Higher-magnification imaging highlights the internal pore structure and the thin SiO_2_ layer covering the conductive silicon framework. Bottom right: schematic illustration of the pore architecture and surface functionalization, showing conductive silicon walls coated with a ∼30–60 nm SiO_2_ layer and further modified with fluorosilane molecules, which render the internal pore surface strongly hydrophobic.

**Figure 3:**
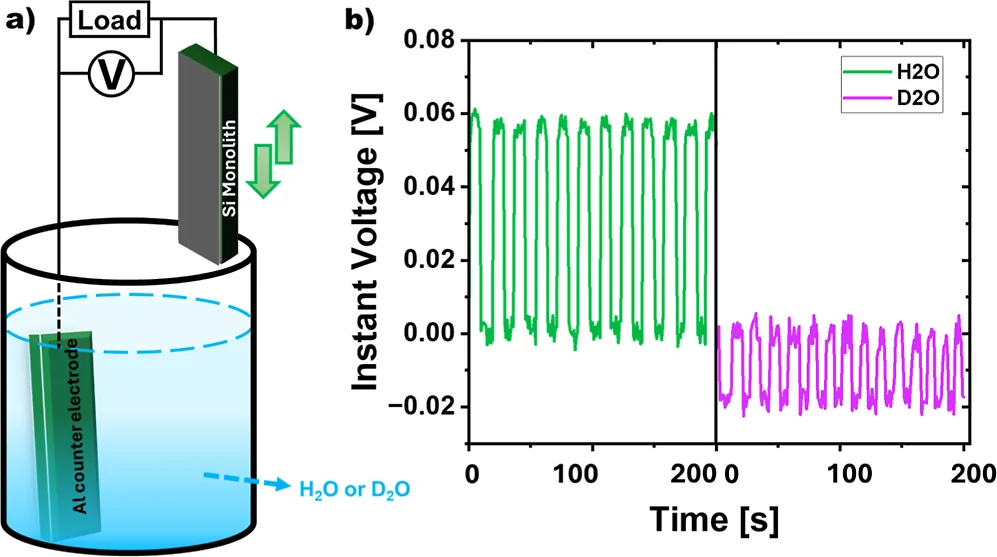
Immersion–emersion electrification of the hydrophobic nanoporous silicon monolith. (a) Schematic illustration of the immersion–emersion measurement configuration, in which the monolith is periodically inserted into and withdrawn from liquid against an immersed counter electrode. (b) Representative voltage output recorded during successive immersion–emersion cycles performed under identical conditions in H_2_O and D_2_O, showing stable oscillatory electrical outputs with liquid-dependent polarity and amplitude.

### 2.3 Droplet Triboelectric Experiments

For the droplet tests, the nanoporous silicon monolith was fixed onto a glass slide and placed at an 80-degree angle. It was then connected to ground via an electrometer (6517B, Keithley Inc.) set to current measurement, which was connected to an oscilloscope (5444D, PicoScope Inc.). The positive electrode was connected to the sample, while the negative - to ground. From a height of 2 cm, deionized (DI) water was dropped onto the sample from a grounded 0.8 mm stainless steel needle as shown in Figure 4a. A constant supply of DI water was supplied using a syringe pump (NE-1000, syringepump.com), with a droplet diameter of 2.1 mm. The velocity of the water droplets was 0.63 m/s, and the experiments were conducted at 34% relative humidity. Latterly, D_2_O was substituted for DI water. The current generated by each drop was recorded using the oscilloscope, and the charge was calculated by integrating the generated charge over time for each drop. The same experiment was used to test the triboelectric response of the PTFE film to DI droplets. The PTFE film was supported by the conductive double-sided adhesive carbon tape (Science Services, MN77817-20) electrode on a glass slide.

**Figure 4:**
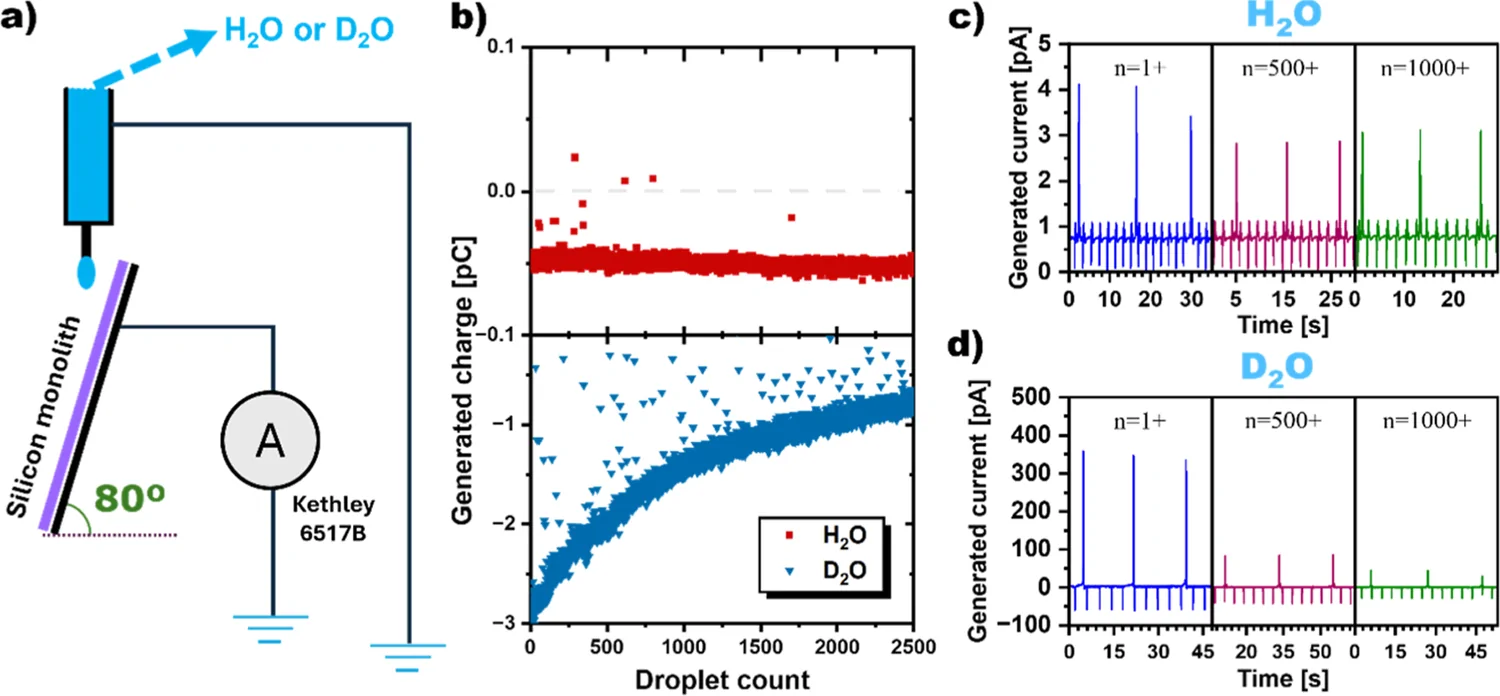
Droplet-based contact electrification of the nanoporous silicon monolith. (a) Experimental configuration for droplet measurements, in which liquid droplets released from a grounded needle periodically contact and detach from the inclined monolith surface while the generated current is recorded. (b) Charge-generation evolution as a function of droplet impact number for H_2_O and D_2_O under identical geometrical and electrical conditions, highlighting distinct stabilization dynamics for the two liquids. Representative current traces for successive (c) H_2_O and (d) D_2_O droplets, exhibiting periodic opposite-polarity events occurring every eighth cycle and fifth cycle, respectively.

### 2.4 Intrusion-Extrusion Triboelectric Experiments

The intrusion-extrusion experiments were performed using a high-pressure variable volume unit (PVT) custom-built by Eurotechnica (HPVV-800) and subsequently modified at CIC energiGUNE. This unit comprises a piston drive module (XL NEMESYS) and a high-pressure steel syringe (10 mL), both supplied by CETONI (Figure 1). The system operates at a maximum pressure of 80 MPa. The drive module is controlled via QmixElements software, which accurately records pressure (±0.1 kPa), volume variation (±0.01 μL), and temperature (±0.01 K) within the cell. During testing, the syringe body was directly connected to a cylindrical stainless steel 304L vessel (Ø 20 mm, length 80 mm) in which samples were placed. This vessel and its fittings were designed and custom-fabricated at CIC energiGUNE (see Figure 1). Water was used as the pressure-transmitting fluid for all intrusion–extrusion triboelectrification tests. The nanoporous silicon monolith, encapsulated in a flexible capsule, was positioned inside the vessel, with a gold wire attached using conductive silver paste. This gold wire was then connected to the external circuit through additional wiring that passed through a custom-built sealing lid. The electrical output (voltage and current) during intrusion–extrusion cycles was recorded using a National Instruments DAQ module (NI 9219).

## 3 Results and Discussion

The solid used in this study is a conductive, hydrophobically grafted nanoporous silicon monolith (Figure 2), whose triboelectric performance and robustness were established in our previous work.^27^ The monolith consists of a p-doped silicon backbone bearing a nanometer-thick SiO_2_ layer, further functionalized with fluorinated alkyl silanes to render the internal pore surface strongly hydrophobic (see Section 2.1). This architecture enables reproducible and efficient solid–liquid contact electrification during pressure-driven intrusion–extrusion cycles while maintaining mechanical integrity. Owing to its electrical conductivity, the monolith acts simultaneously as the triboelectric active material and the charge collector, delivering substantially higher energy per cycle and improved charge collection compared to powder-based porous systems^20^ and ensuring stable operation through repeated cycling.

### 3.1 Immersion–Emersion Test Results

During periodic immersion–emersion of the monolith in bulk liquid (Figure 3a), distinct electrification responses are observed for H_2_O and D_2_O (Figure 3b). Repeated immersion–emersion cycles in H_2_O generate stable, reproducible voltage oscillations with a consistent polarity and amplitude over successive cycles (see Section 2.2). When the solid is emersed, the instantaneous voltage is ∼0 V, and upon immersion it takes a positive value of ∼0.059 ± 0.001 V with respect to a reference electrode that remains immersed in the same liquid. Then, when D_2_O replaces H_2_O under otherwise identical conditions, the electrical signal exhibits a markedly different response. The signal polarity is inverted with respect to H_2_O, with voltage taking negative values with respect to the Al reference electrode upon immersion of the monolith. Meanwhile, the signal amplitude is reduced to ∼0.020 ± 0.001 V.

These results demonstrate that isotopic substitution alone is sufficient to qualitatively alter the electrification response when wetting is limited to surface nanoroughness. Notably, a previous study employing a U-tube SL-TENG, reported similar reduction of output upon replacing H_2_O with D_2_O.^22^ On the contrary, the polarity inversion observed in Figure 3 suggests that isotope effects are strongly configuration dependent. Therefore, this comparison indicates that even among seemingly similar configurations of SL-TENG, the specific wetting protocol can critically determine how the isotope-dependent effects emerge.

### 3.2 Droplet Test Results

In the droplet-based electrification experiments (Figure 3a), the nanoporous silicon monolith exhibits markedly different charge-generation behavior when exposed to H_2_O and D_2_O droplets under identical geometrical and electrical conditions (see Section 2.3). For H_2_O, successive droplets produce a strong initial electrical response that rapidly approaches a steady state, with the charge generated per droplet remaining nearly constant after a relatively small number of impacts (Figure 4b), with an average value of −0.020 ± 0.001 pC. In contrast to the polarity inversion observed in immersion–emersion experiments, although both H_2_O and D_2_O droplets generate signals with negative charge polarity, their temporal evolution differs substantially. D_2_O droplets produce a significantly larger initial charge, followed by a pronounced decay in signal magnitude with increasing droplet number, eventually reaching a quasi-saturated regime at significantly lower charge per droplet. Despite this decay, the charge generated per droplet remains more than 1 order of magnitude higher than in the H_2_O case. The current signals shown in Figure 4 further illustrate the distinct dynamics of the two liquids. For H_2_O (Figure 4c), the current response displays an alternating character, with peaks extending on both sides of the baseline. In contrast, the D_2_O signal (Figure 4d) is strongly asymmetric: the recurrent peaks are exclusively negative, with positive counterparts largely absent, and progressively decrease with droplet number (for further details, see Supp. Inf. Section S4). These differences in symmetry, sign distribution, and decay behavior demonstrate that transient, repeatedly renewed wetting leads to fundamentally distinct charging dynamics for H_2_O and D_2_O, despite identical surface chemistry and contact geometry.

In addition, both liquids exhibit occasional large-amplitude spikes; however, their distribution differs. For H_2_O droplets (Figure 4c), pronounced current peaks of opposite polarity appear periodically at every eighth drop impact, whereas for D_2_O droplets (Figure 4d), similar events occur every fifth drop impact. The absence of such periodic events for the PTFE film used for reference comparison (see Supp. Inf. Section S5)^28^ further suggests that the nanoporous architecture enables localized charge accumulation and intermittent discharge processes not accessible in compact solid films, indicating a morphology-dependent effect. It is fascinating that such periodic discharges were not observed in immersion-emersion tests (Figure 3) nor in U-tube TENG.^22^

As a preliminary stability check after the droplet-testing campaign, the contact angle of the monolith was measured again, and no significant difference was observed compared with the initial value. In addition, complementary H_2_O droplet experiments performed approximately two months after the original measurements showed that the polarity of both the regular charge peaks and the additional discharge peaks remained consistent with the original droplet experiments during 10 min of operation.

### 3.3 Intrusion–Extrusion Test Results

In the pressure-driven intrusion–extrusion experiments (Figure 5a), each intrusion event generates a marked current and voltage response, followed by an opposite-polarity signal upon extrusion (Figure 5b,c), reflecting the reversible expansion and contraction of the solid–liquid interfacial area under confinement (see Section S4). Surprisingly, in contrast to the immersion–emersion and droplet tests, substitution of H_2_O with D_2_O does not change the polarity of electrification, similarly to U-tube TENG experiments;^22^ instead, it significantly affects their magnitudes. Under identical pressure protocols, D_2_O consistently produces higher peak and sustained voltages and currents, with representative voltage peak values of 94 ± 4 mV for H_2_O and 219 ± 3 mV for D_2_O, resulting in substantially higher instantaneous power density and electrical energy per cycle than H_2_O (Figure 5d). Fascinatingly, this effect is opposite to U-tube TENG experiments, where a reduction of the generated signal was reported upon substitution of H_2_O with D_2_O.^22^ Together with the immersion–emersion and droplet results, these findings demonstrate that forced wetting of nanopores constitutes a distinct electrification regime, in which isotopic substitution leads to trends that are not observed when wetting is limited to surface roughness or transient droplet contact. Of course, pressure-driven intrusion–extrusion supplies substantially higher and quantifiable mechanical work, potentially activating defect-mediated interfacial processes associated with intrusion–extrusion contact electrification,^27^ that are energetically inaccessible under immersion–emersion or droplet conditions.

**Figure 5:**
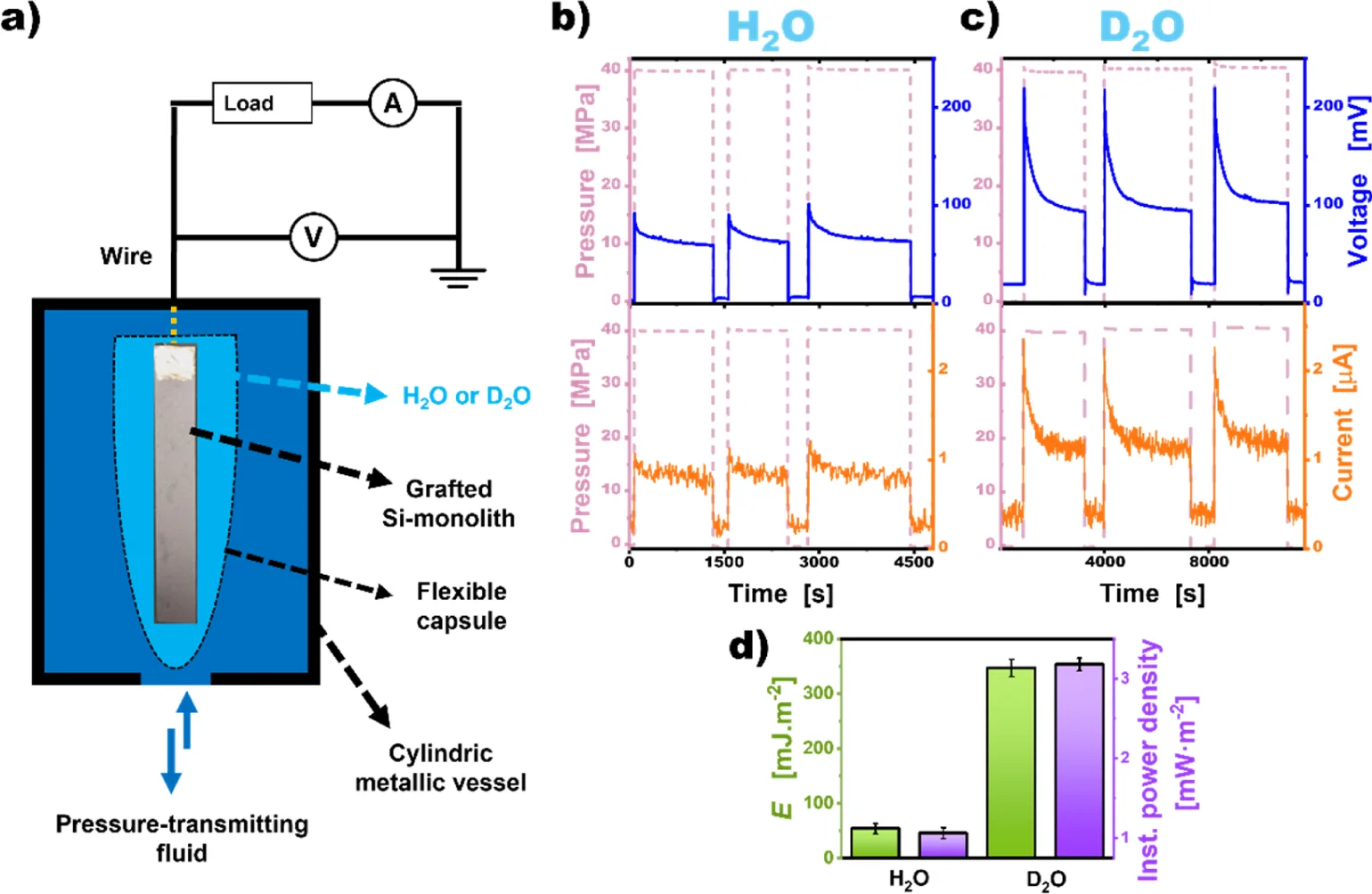
Pressure-driven intrusion–extrusion electrification in hydrophobic nanopores. (a) Schematic representation of the high-pressure setup used to force liquid intrusion into and extrusion from the nanoporous monolith while recording electrical output. Representative voltage and current signals obtained during intrusion and extrusion cycles for (b) H_2_O and (c) D_2_O. (d) Comparison of electrical performance, showing energy generated per cycle and instantaneous power density measured for H_2_O and D_2_O under identical pressure protocols. The instantaneous power density was calculated from the peak amplitude of the electrical signal by multiplying the measured voltage and current values.

### 3.4 Discussion

We emphasize that the discussion presented below should be viewed as an invitation to further investigation of the observed phenomena rather than as definitive conclusions on the underlying mechanism. The observation that isotope substitution produces distinct responses across all explored wetting regimes demonstrates that H_2_O/D_2_O comparison provides an additional experimental dimension for probing protocol-dependent contact-electrification mechanisms beyond changes in material chemistry or device architecture.

Isotope substitution modifies the nuclear dynamics of water without changing its electronic structure.^29^ Experimental neutron scattering and simulation studies show that heavy water possesses a slightly more tetrahedral and more structured hydrogen-bond network than light water.^30^ Nuclear quantum effects associated with the lighter proton increase zero-point motion in H_2_O, which modifies hydrogen bonding through competing effects, including partial destabilization of the hydrogen-bond network and changes in intermolecular interactions.^29^ These differences are reflected in the dynamical properties of the liquids: heavy water exhibits lower self-diffusion coefficients and a viscosity approximately 20% higher compared to H_2_O.^31^ In addition, vibrational frequencies depend on nuclear mass through zero-point energy effects, resulting in a systematic shift of both stretching and bending modes to lower frequencies for O–D relative to O–H vibrations,^32,33^ which results in a weaker hydrogen bond in H_2_O vs D2O.^34^ Below, we provide several hypotheses that may reflect these differences in terms of performed electrification experiments.

#### 3.4.1 Proton vs Deuteron Transport (Grotthuss-Like Mechanism)

Proton hopping along hydrogen-bond networks is known to be significantly faster than deuteron transport, reflecting a strong kinetic isotope effect in aqueous systems.^35^ In electrochemical devices, replacing H_2_O with D_2_O leads to reduced ionic conductivity, slower mass transport, and increased overpotentials (on the order of ∼190 mV at 1 A cm^–2^), indicating slower charge transport kinetics in heavy water.^35^ These differences are generally attributed to higher activation barriers for bond breaking, stronger hydrogen bonding, and reduced mobility of D^+^ compared to H^+^.^35^ Because low-ion water was used in both cases, differences arising from added electrolyte impurities are expected to be minimized; however, intrinsic isotope-dependent differences in autoionization and proton/deuteron mobility may still contribute to the observed electrification response.

At interfaces, such differences in ionic mobility can directly affect charge separation processes. A more mobile proton population in H_2_O can be redistributed more rapidly during contact, whereas slower deuteron transport in D_2_O may hinder such equilibration and alter the relative contributions of different charge carriers.

A particularly relevant example is provided by nanoconfined water films between graphene and mica, where proton permeation through graphene leads to the formation of interfacial H^+^–OH^–^ ion pairs spanning the interface.^36^ These ion pairs can effectively “clamp” the interface and strongly influence interfacial mobility and friction. In contrast, graphene is significantly less permeable to deuterons, which suppresses this mechanism in D_2_O and results in markedly different interfacial dynamics despite nearly identical electronic structure of the liquid.^36^

Within the context of the present work, these isotope-dependent transport effects can be related differently to the three electrification regimes. In immersion–emersion experiments, where wetting is limited to surface nanoroughness and interfacial charge exchange is likely governed by near-surface ion transfer, suppression of fast proton transport in D_2_O may provide a plausible explanation for both the reduced electrification magnitude and the observed polarity inversion. In this case, the balance between proton transfer and competing processes (e.g., hydroxide adsorption or electron transfer) may shift when moving from H_2_O to D_2_O, leading to a change in dominant charge carrier and thus sign reversal.

Similarly, the reduced electrification observed in the U-tube SL-TENG configuration^22^ upon replacing H_2_O with D_2_O, is consistent with this interpretation, as these systems also likely rely on interfacial ion transport under relatively weak confinement and limited mechanical forcing.

In contrast, this mechanism cannot directly explain the behavior observed in droplet-based and intrusion–extrusion experiments. In droplet experiments (Figure 4), D_2_O produces significantly larger initial electrification despite slower ion transport, which is inconsistent with a simple proton-mobility-controlled picture. Likewise, in pressure-driven intrusion–extrusion, D_2_O generates higher electrical output while preserving polarity, indicating that charge generation is not limited by proton transport kinetics alone under these conditions. These regimes involve either transient nonequilibrium wetting (droplets) or forced nanoconfinement with substantial mechanical work input (intrusion–extrusion), where additional mechanisms such as defect-mediated processes^27^ or interfacial structuring likely dominates.

#### 3.4.2 Stronger Hydrogen-Bond Network and Interfacial Orientation Effects

Isotope substitution leads to a more structured hydrogen-bond network in D_2_O compared to H_2_O, with increased tetrahedral order and modified local coordination.^37,38^ These differences are expected to influence not only bulk structure but also interfacial organization.

Phase-sensitive Sum-Frequency Generation spectroscopy (SFG) studies show that H_2_O and D_2_O interfaces exhibit measurable differences in spectral response, reflecting isotope-dependent intermolecular interactions and modified orientational distributions of interfacial molecules.^39^ These differences indicate changes in interfacial polarization, although they do not demonstrate a complete inversion of dipole orientation, which might have explained the polarity change in immersion-emersion experiments here. However, SFG measurements at oxide–water interfaces demonstrate that interfacial water orientation is highly sensitive to local conditions and can undergo flipping depending on surface charge, with water molecules reversing their average orientation across the point of zero charge^40^ This establishes that relatively small perturbations of the hydrogen-bonding environment can lead to significant changes in interfacial dipole orientation, which may have both quantitative and qualitative differences in contact electrification.

#### 3.4.3 Defect Stabilization and Slower Relaxation in D_2_O

In intrusion–extrusion experiments, electrification is associated with the formation of interfacial grafting defects during forced wetting and subsequent extrusion.^27^ Isotope substitution may provide a plausible mechanism for enhancing this effect, as D_2_O exhibits slower hydrogen-bond rearrangement and molecular reorientation dynamics than H_2_O because nuclear quantum effects are weaker for deuterium.^32^ In addition, D_2_O forms a slightly more structured and stronger hydrogen-bond network.^38^ As a result, defects created during intrusion–extrusion may relax more slowly in D_2_O than in H_2_O. This kinetic stabilization of nonequilibrium configurations can lead to longer-lived charge separation and reduced recombination, thereby increasing the measured electrification. This mechanism is consistent with the higher electrification observed for D_2_O in intrusion–extrusion experiments, while contrasting with surface-limited regimes (immersion–emersion (Figure 3) and U-tube TENG^22^) where ion mobility may dominate.

Moreover, from recent reports, it can be seen that the intrusion of D_2_O into hydrophobic nanopores leads to higher intrusion pressure compared to H_2_O,^41,42^ which results in the overall larger mechanical energy input, which, in turn, may lead to a high number of defect formation and hence larger observed electrification.

In droplet-based electrification, the interface is repeatedly created and destroyed under highly nonequilibrium conditions, which fundamentally differs from both immersion–emersion and intrusion–extrusion regimes. In such transient wetting, charge generation is governed not only by initial contact but also by charge accumulation and incomplete discharge over successive impacts. Previous studies have shown that moving or impacting water droplets can progressively charge solid surfaces, with charge build-up and intermittent discharge events depending on interfacial relaxation and transport processes.^28^ Furthermore, recent studies on liquid slide electrification further support this interpretation.^43–46^ Moving water droplets on hydrophobic surfaces have been shown to spontaneously separate charge at the receding contact line, leaving surface charge behind and producing history-dependent electrostatic effects. Importantly, this charge accumulation can modify droplet motion, apparent wettability, and the interaction between the contact line and surface defects, so that successive droplets do not necessarily interact with an electrically neutral or unchanged surface. In our droplet experiments, this provides a plausible framework for understanding why repeated H_2_O and D_2_O impacts lead to distinct charge-evolution profiles and intermittent opposite-polarity discharge events: the measured signal may reflect not only the instantaneous droplet–surface contact, but also the evolving charge state of the nanoporous hydrophobic interface.

Within this context, the larger electrification observed for D_2_O droplets may be rationalized by slower relaxation dynamics and reduced charge dissipation compared to H_2_O. Because D_2_O exhibits slower hydrogen-bond rearrangement and molecular reorientation, as well as reduced ionic mobility, charges generated during each droplet impact are expected to persist longer at the interface. This can lead to cumulative charge build-up and enhanced signal magnitude, consistent with the higher initial charge and slower decay observed experimentally. The strongly asymmetric current response and periodic discharge events observed for D_2_O further suggest that charge accumulation reaches a threshold before being released intermittently, a behavior that is characteristic of systems with hindered charge relaxation.

In contrast, faster relaxation and charge redistribution in H_2_O promote more efficient discharge between successive droplets, resulting in lower net electrification and more symmetric current signals. Thus, the isotope-dependent differences observed in droplet experiments may be understood as a consequence of transient wetting combined with kinetically limited charge dissipation, rather than equilibrium ion availability or steady-state interfacial structure.

## 4 Conclusions

This work provides, to our knowledge, the first systematic experimental comparison of solid–liquid electrification for H_2_O vs D_2_O using the same solid material across three different regimes: droplet-based contact, immersion–emersion, and pressure-driven intrusion–extrusion. While isotope effects have been widely exploited in studies of hydrogen bonding, electrokinetics, and interfacial dynamics, their role as a comparative isotopic tool in contact electrification remains extremely limited. By showing that simple isotopic substitution can qualitatively and quantitatively alter the electrification response in a regime-dependent manner, this study demonstrates that electrification responses obtained under different wetting conditions cannot be interpreted interchangeably.

From a broader perspective, the H_2_O/D_2_O comparison introduced here may serve as a useful experimental probe for identifying protocol-dependent differences in solid–liquid contact electrification, while leaving the detailed microscopic mechanisms as an important subject for future studies. By combining (i) a single, well-defined solid platform, (ii) multiple electrification regimes that span surface wetting to full pore intrusion, and (iii) isotopically distinct but chemically equivalent liquids, this approach enables the community to benchmark electrification responses across fundamentally different interfacial states. Such a framework is particularly valuable in a field where disparate experimental geometries are often discussed under a common conceptual umbrella. Beyond the specific case of H_2_O and D_2_O, the present results illustrate how controlled perturbations of interfacial dynamics (rather than changes in material chemistry) can reveal hidden nonequivalences between electrification regimes, providing a robust experimental basis for future interpretation, comparison, and modeling of solid–liquid contact electrification.

## Supplementary Material



## Notes

Contact-angle measurements of H_2_O and D_2_O on the silicon monolith (Section S1); Pore size distribution of the monolith (Section S2); System validation using a reference electrode in immersion-emersion tests (Section S3); Evolution of triboelectric droplet experiments (Section S4) and Triboelectric droplet experiments with PTFE film (Section S5) (PDF)
