# Supplementary material for: Distinct Electrification Regimes at Solid–Liquid Interfaces Revealed by Water Isotope Substitution
Source: J Phys Chem C Nanomater Interfaces. 2026 Jul 28;130(36):12748–56. doi: 10.1021/acs.jpcc.6c04134 (PMC13573812; doi:10.1021/acs.jpcc.6c04134)
Supplement: Supplementary file 1 [file jp6c04134_si_001.pdf]

Distinct Electrification Regimes at Solid–Liquid Interfaces Revealed by Water Isotope Substitution

Mesude Z. Arkan,[1,†] *Luis Bartolomé,*[2,†] *Raivis Eglītis,*[3] *Manuel Brinker,*[4,5] *Eder Amayuelas,*[2] *Patrick Huber,*[4,5,6] *Mirosław Chorążewski,*[1] *Simone Meloni,*[7,*] *Andris Šutka,*[3,*] *Yaroslav Grosu,*[1,2,*]

[1]Institute of Chemistry, University of Silesia, 40-006 Katowice, Poland

[2]Centre for Cooperative Research on Alternative Energies (CIC energiGUNE), Basque Research and Technology Alliance (BRTA), Alava Technology Park, Albert Einstein 48, 01510 Vitoria-Gasteiz, Spain.

[3]Institute of Physics and Materials Science, Faculty of Natural Sciences and Technology, Riga Technical University, P. Valdena Street 3/7, Riga LV-1048, Latvia

[4]Hamburg University of Technology, Institute for Materials and X-Ray Physics, Denickestr. 15, 21073, Hamburg, Germany

[5]Centre for X-ray and Nano Science CXNS, Deutsches Elektronen-Synchrotron DESY, Notkestr. 85, 22607, Hamburg, Germany

[6]Centre for Molecular Water Science CMWS, Deutsches Elektronen-Synchrotron DESY, Notkestr. 85, 22607, Hamburg, Germany

[7]Dipartimento di Scienze Chimiche e Farmaceutiche Università degli Studi di Ferrara, Via Luigi Borsari 46, I-44121 Ferrara, Italy.

* To whom correspondence should be addressed; E-mails: simone.meloni@unife.it, andris.sutka@rtu.lv, ygrosu@cicenergigune.com

† These authors contributed equally

Table of contents:

Supporting Section 1: Contact-angle measurements of H_2O and D_2O on the silicon monolith

Supporting Section 2: Pore size distribution of the monolith

Supporting Section 3: System validation using a reference electrode in immersion-emersion tests

Supporting Section 4: Evolution of triboelectric droplet experiments

Supporting Section 5: Triboelectric droplet experiments with PTFE film

Supporting References

S1. Contact-angle measurements of H_2O and D_2O on the silicon monolith:

To evaluate whether isotope substitution modifies the macroscopic wettability of the hydrophobic nanoporous silicon monolith, apparent contact-angle measurements were performed using H_2O and D_2O droplets on the monolith surface. As shown in Figure S3, both liquids exhibited very similar contact angles, with values of approximately 129–130° for H_2O and 127–130° for D_2O. These results indicate that, at the macroscopic level, H_2O and D_2O present comparable wettability on the hydrophobic fluorinated surface.

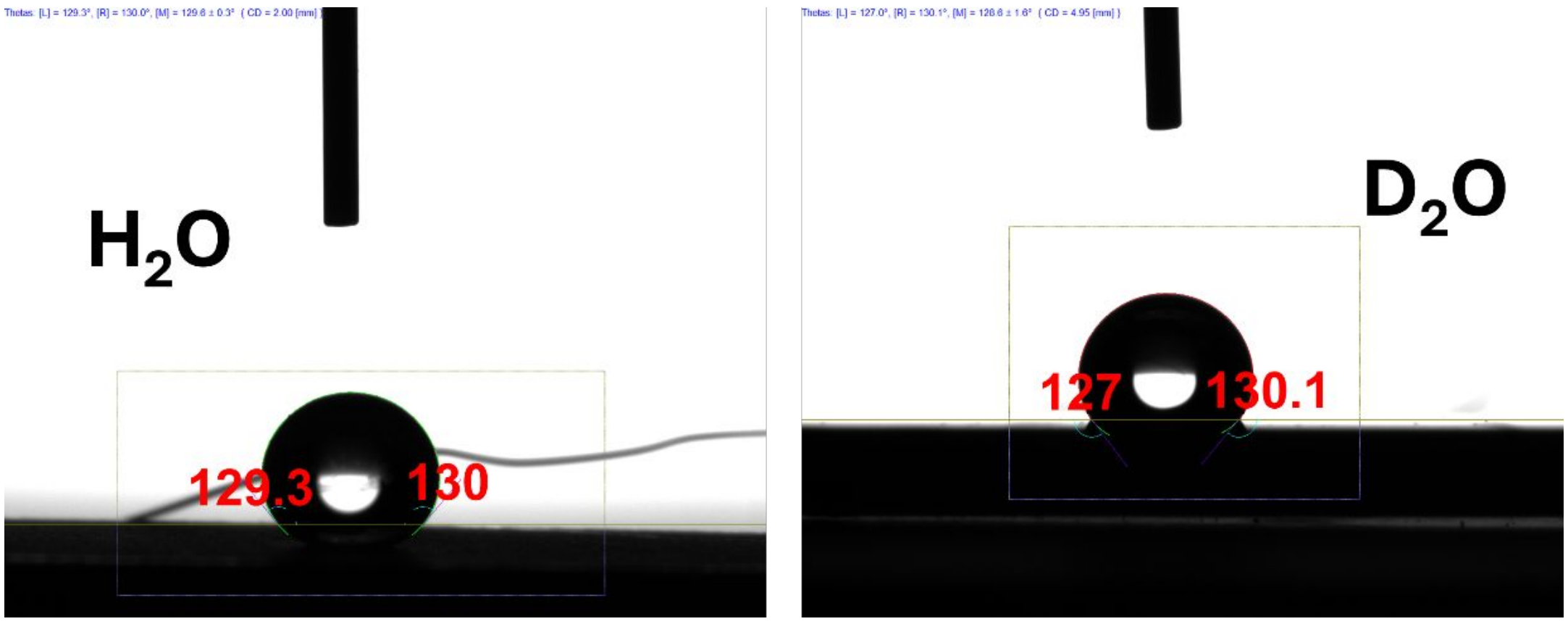

Figure S1. Contact-angle measurements of H_2O and D_2O on the hydrophobic silicon monolith. Apparent contact angles measured for H_2O and D_2O droplets on the hydrophobic surface of the nanoporous silicon monolith, showing comparable macroscopic wettability for both liquids.

S2. Pore size distribution of the monolith:

The pore size distribution was determined from nitrogen adsorption measurements using the BJH model and is centred around ~15 nm.

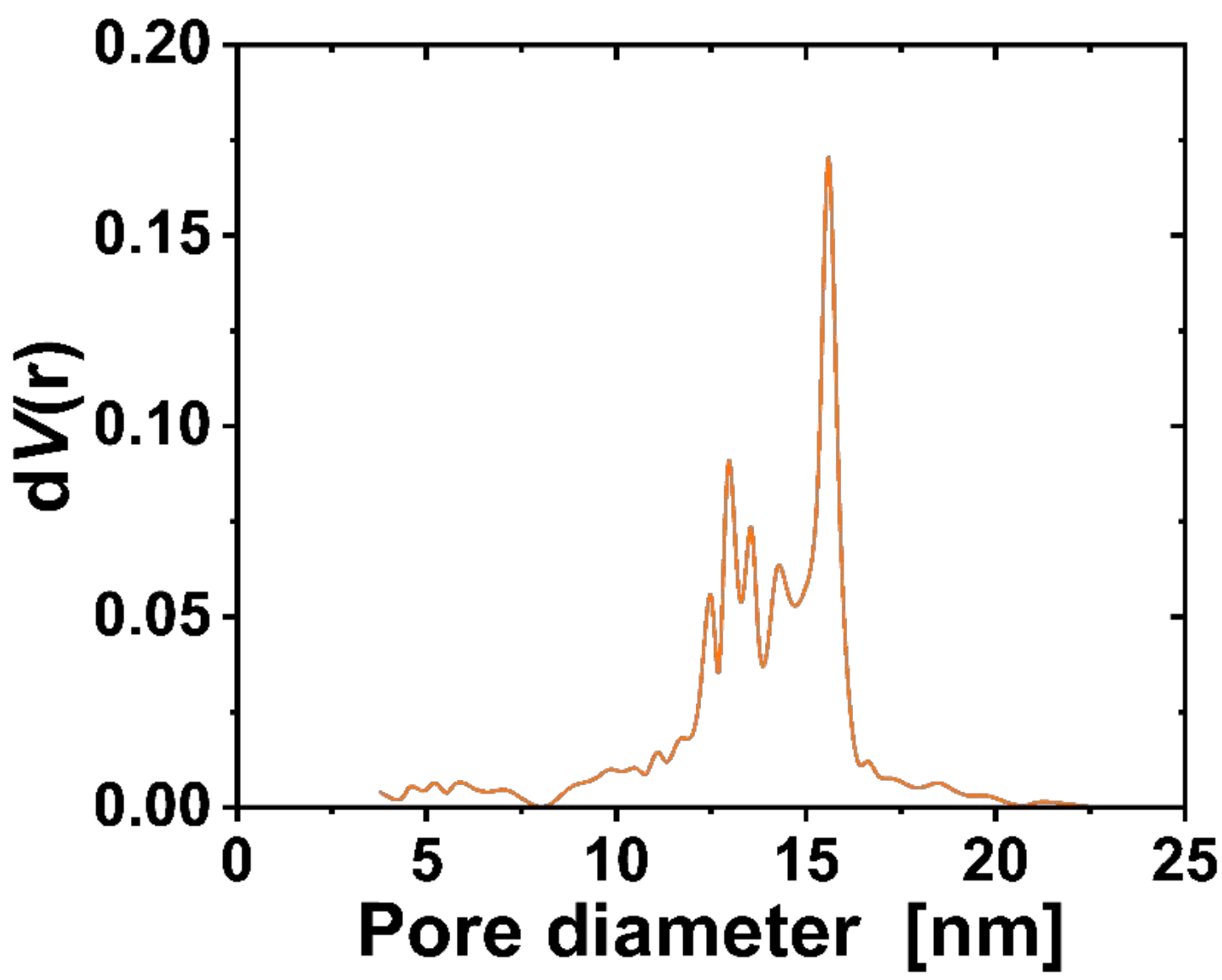

Figure S2. Pore size distribution of silicon monolith. Pore size distribution of the silicon monolith determined by N_2 adsorption measurements using the BJH model.

S3. System validation using a reference electrode in immersion-emersion tests:

Prior to the experiments with the nanoporous silicon monolith, the experimental setup was validated using a conventional triboelectric configuration consisting of a PTFE-coated aluminum electrode and pure H_2O. The measurements were performed under the same geometrical and electrical conditions as those used in the main experiments. This preliminary test confirmed the proper operation of the setup, yielding stable and reproducible voltage signals characteristic of solid–liquid contact electrification (see Figure S4). These results serve as a reference.

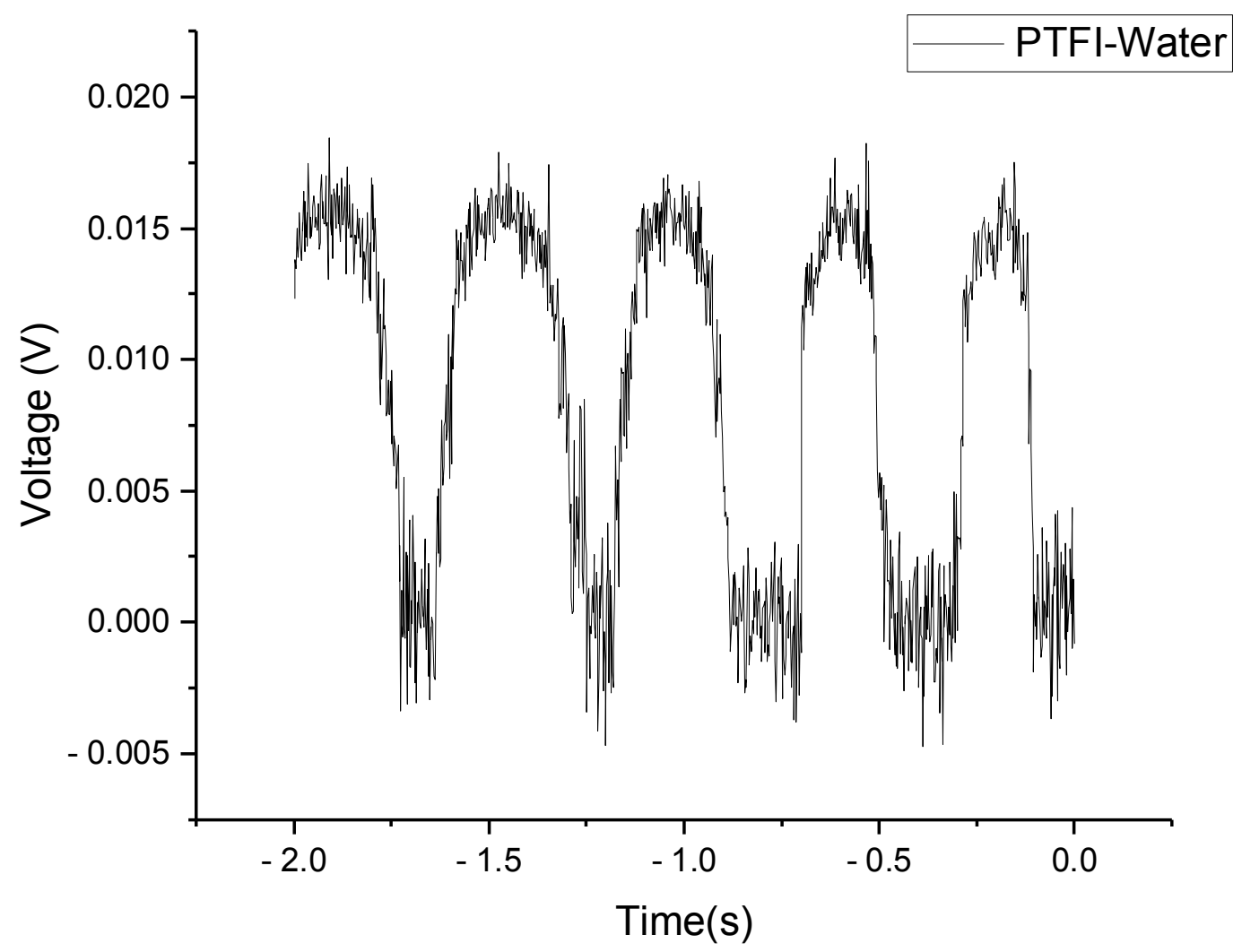

Figure S3. Validation of the experimental setup using a PTFE-coated aluminum electrode. Voltage signal obtained using a PTFE-coated aluminum electrode with H_2O.

S4. Evolution of triboelectric droplet experiments:

The evolution of the droplet-based electrification signal was monitored over a total of 2500 impact events (Figure S1A), corresponding to the full duration of the experiment. Within this interval, approximately 400 additional positive discharge peaks were observed (see Figure S1B). At the early stages of the experiment, these additional discharge events exhibited a pronounced difference between the two liquids: the peak charge associated with D_2O droplets was nearly two orders of magnitude higher than that measured for H_2O (40.5 pC for D_2O compared to 0.455 pC for H_2O). As the experiment progressed, this disparity gradually decreased. After approximately 300 such discharge events, the difference between the two systems was reduced to roughly one order of magnitude, with peak charges of 4.21 pC and 0.50 pC recorded for D_2O and H_2O, respectively.

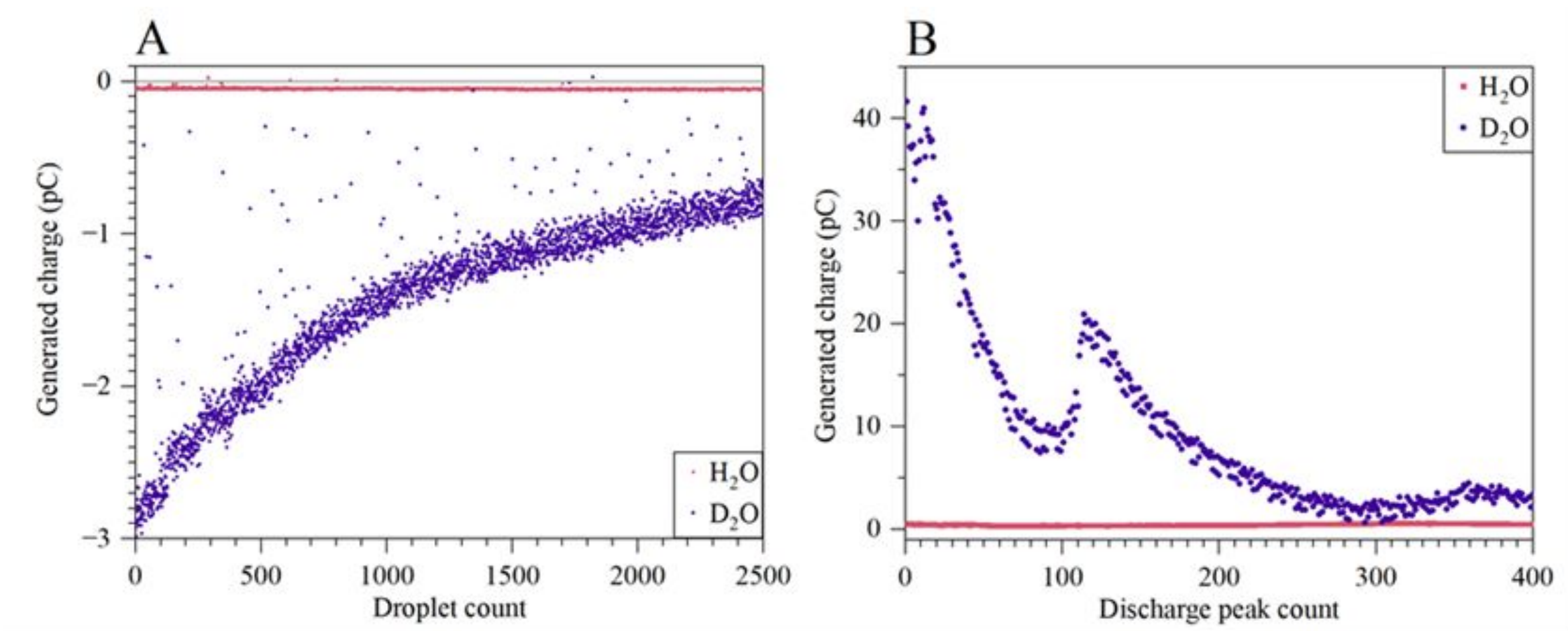

Figure S4. Charge-generation evolution as a function of droplet impact number for H_2O and D_2O. (A) Charge-generation evolution as a function of droplet impact number for H_2O and D_2O under identical geometrical and electrical conditions for the 2500 peaks of the experiment; only the regular peaks are shown. (B) The periodically positive peaks (around 400) that occur during the whole experiment, where the difference between H_2O and D_2O gradually lessens.

S5. Triboelectric droplet experiments with PTFE film:

Figure S2 shows the current generated during repeated contact–separation events between a water droplet and a flat PTFE film, serving as a reference measurement for comparison with the porous monolith samples discussed in the main text. Prior to data acquisition, the system was stabilised by depositing 1000 droplets onto the surface in order to reach a steady electrostatic state. Such preconditioning is commonly employed in droplet-based contact electrification experiments on hydrophobic fluoropolymer surfaces, where repeated droplet interactions lead to progressive surface charge accumulation and signal stabilisation, as previously reported in the literature.[1]

After stabilisation, the generated current was recorded for 30 consecutive droplet cycles. The PTFE film exhibits reproducible current transients with consistent polarity throughout the measurement, and no periodic polarity inversion is observed. This behaviour contrasts with that of the porous monolith samples (Figure 2), indicating that the alternating polarity response described in the main manuscript is not an intrinsic feature of the liquid–solid material combination or measurement protocol, but instead arises from morphology-dependent effects associated with porous structures.

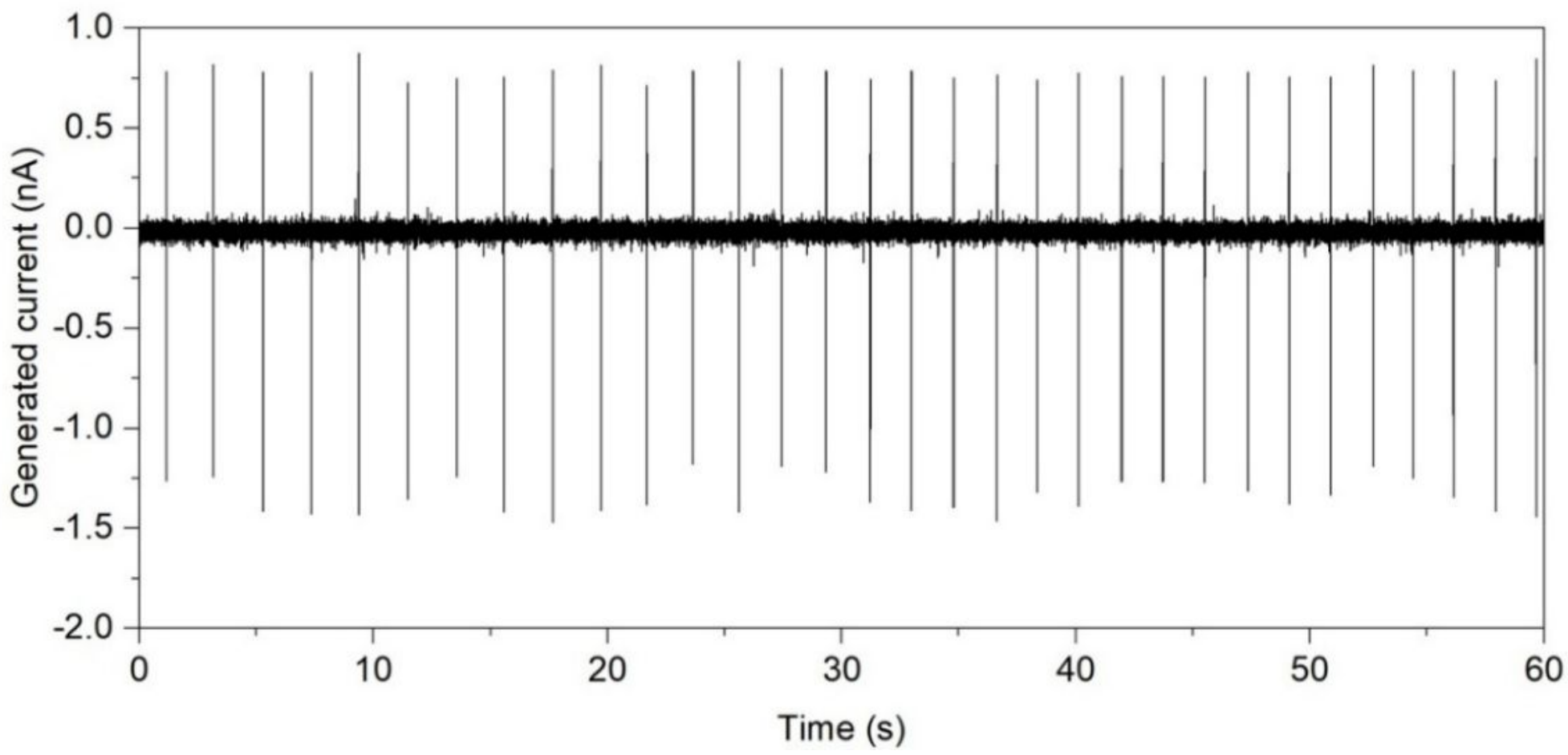

Figure S5. Droplet-based contact electrification of a PTFE film. Current generated during contact separation of a water droplet with the PTFE surface for 30 subsequent cycles. Before measurement, the sample was stabilised by dripping 1000 droplets.[1]

References

(1) Stetten, A. Z.; Golovko, D. S.; Weber, S. A. L.; Butt, H.-J. Slide Electrification: Charging of Surfaces by Moving Water Drops. *Soft Matter* **2019**, *15* (43), 8667–8679. https://doi.org/10.1039/C9SM01348B.
